# Artesunate-amodiaquine efficacy in Congolese children with acute uncomplicated falciparum malaria in Brazzaville

**DOI:** 10.1186/1475-2875-12-53

**Published:** 2013-02-05

**Authors:** Mathieu Ndounga, Pembe Issamou Mayengue, Prisca Nadine Casimiro, Dieudonné Loumouamou, Leonardo K Basco, Francine Ntoumi, Philippe Brasseur

**Affiliations:** 1 Unité de Recherche sur le Paludisme, Centre d’Etudes sur les Ressources Végétales (CERVE), Brazzaville BP 1249, République du Congo; 2 Fondation Congolaise pour la Recherche Médicale (FCRM), Brazzaville, BP 2672, République du Congo; 3 Faculté des Sciences de la Santé, Université Marien Ngouabi, Brazzaville, BP 2672, République du Congo; 4 Circonscription Socio-Sanitaire de Makélékélé, Ministère de la Santé, Brazzaville, République du Congo; 5 Institut de Recherche pour le Développement (IRD), Unité Mixte de Recherche 198, Unité de Recherche des Maladies Infectieuses et Tropicales Emergentes (URMITE), Faculté de Médecine La Timone, Université Aix-Marseille, Marseille, France; 6 Laboratoire de Recherches sur le Paludisme, Organisation de Coordination pour la lutte contre les Endémies en Afrique Centrale (OCEAC), Yaoundé, Cameroon; 7 Institut de Recherche pour le Développement (IRD) Unité mixte de Recherche 198, Dakar, BP 1386, Sénégal

**Keywords:** Drug resistance, Artemisinin, Combination therapy, Chloroquine, Drug efficacy, Congo-Brazzaville

## Abstract

**Background:**

Congo-Brazzaville adopted artemisinin-based combination therapy (ACT) in 2006. Artesunate-amodiaquine (AS + AQ) and artemether-lumefantrine are the first-line and second-line anti-malarial drugs to treat uncomplicated *Plasmodium falciparum* malaria, respectively. The baseline efficacy of AS + AQ was evaluated from February to August 2005 in patients living in Brazzaville, the capital city of the Republic of Congo.

**Methods:**

One hundred and ninety-seven patients (96 ≤5 years old and 101 >5 years old, including adults) were recruited in a non-randomized study, treated under supervision with AS + AQ, and were followed up for 28 days in accordance with the 2003 World Health Organization protocol. *Plasmodium falciparum* recrudescent isolates from day 7 to day 28 were compared to pretreatment isolates by polymerase chain reaction (PCR) to distinguish between re-infection and recrudescence.

**Results:**

The overall efficacy of AS + AQ after PCR correction on day 28 was 94.4%. An adequate clinical and parasitological response was observed in 94.3% and 94.4% of children aged ≤5 years old and those aged >5 years old (including adults), respectively. The main reported adverse events were dizziness, vomiting, diarrhoea, pruritus, headache, anorexia, and abdominal pain.

**Conclusion:**

This study has shown the high efficacy of AS + AQ in Congolese patients of all ages with acute uncomplicated falciparum malaria and serves as the baseline efficacy and tolerance of this ACT in Brazzaville.

## Background

In 2006, there were 247 million malaria cases in 109 endemic countries, of which 212 million cases were registered in Africa. Mortality due to malaria was estimated to be 881,000 deaths, of which 91% occurred in Africa, mostly in children aged <5 years old. The Republic of Congo reported 1,331,668 malaria cases and 4,566 deaths associated with malaria in 2006, while in 2009, 92,855 cases and 116 deaths due to malaria were reported [1,2].

The end of the 20th Century was marked by a considerable increase in global malaria burden due to resistance of the parasites to anti-malarial drugs, in particular to chloroquine [3-6]. However, since 2000, an international deployment of artemisinin-based combination therapy (ACT) has contributed to the reduction of malaria prevalence in several countries [1,2,7-9]. In 2004, 25 African countries adopted ACT for the first-line treatment of uncomplicated malaria [10], and the Republic of Congo adopted the new anti-malarial drug policy in 2006 [11]. By 2008, 33 of 43 African countries had adopted ACT [2].

Before implementation of ACT in the Republic of Congo, malaria was the third cause of medical consultation [12]. In the health centres of Brazzaville, the capital city of the Republic of Congo, 22 to 45.7% of febrile patients were infected with malaria parasites [13]. In the Department of Paediatrics of the main hospital of Brazzaville, Centre Hospitalo-Universitaire de Brazzaville, malaria represents one of the major causes of hospitalization and death [14-16].

In Congo, chloroquine, the former first-line anti-malarial drug, was shown to have a very low efficacy, with a failure rate ranging from 43 to 62.5% on day 14 and 96% on day 28 [17,18]. All *Plasmodium falciparum* isolates collected and analysed in these studies carried K76T substitution in *P. falciparum* chloroquine resistance transporter (*pfcrt*) gene, which is associated with chloroquine resistance. Sulphadoxine-pyrimethamine, the former alternative drug to treat chloroquine-resistant malaria, had also shown low efficacy, with day-28 failure rate of 30% [19]. Analysis of dihydrofolate reductase (*dhfr*) and dihydropteroate synthase (*dhps*) genes in clinical *P. falciparum* isolates has demonstrated a high prevalence of ‘quadruple’ mutations consisting of *dhfr* triple mutation (N51I, C59R, S108N) and *dhps* mutation A437G [20,21].

In 2006, the Republic of Congo adopted artesunate + amodiaquine (AS + AQ) and artemether-lumefantrine as first-line and second-line drugs, respectively, for the treatment of acute uncomplicated malaria. Only one study had evaluated the efficacy of these combinations in a rural area in Congo [22]. Although the AS-AQ coformulation is available in Africa today, until 2009 AS and AQ were only available as separate drug components. In the present non-randomized study, data on AS + AQ (Arsucam^®^) efficacy in 2006 in Brazzaville are presented.

## Methods

### Study zone

The total population of the Republic of Congo was 3,697,490 inhabitants in 2007, and 1,373,382 of these persons (37%) reside in Brazzaville, the capital city [23]. Brazzaville is divided into seven districts: Bacongo, Makelekele, Poto-Poto, Moungali, Ouenze, Talangaï, and Mfilou. The present study was conducted in Makelekele district where 298,292 inhabitants live in urban and suburban areas. Patients were enrolled in Terinkyo urban health centre, located in the urban area of Makelekele district and Madibou health centre, located in the suburban area of the district. Previous malaria surveillance from 2003 to 2007 showed that 22.3% of febrile patients consulting Tenrikyo health centre and 44.7% of febrile patients seen at the Madibou health centre were infected with malaria parasites [13]. In the urban area of Makelekele district, malaria is hypo- to meso-endemic, while in its suburban area, malaria is hyperendemic [24,25].

### Patients

All febrile patients were referred to the health centres’ laboratory for malaria parasite screening. Giemsa-stained thick blood films were prepared from finger-prick capillary blood and examined under the microscope. Symptomatic patients with at least 2,000 asexual parasites/μL were examined by a physician. Patients with the following criteria were included in the study: (i) mono-infection with *P. falciparum* ≥2,000 asexual parasites/μL, (ii) axillary temperature >38°C, (iii) absence of danger signs in young children (unable to drink or eat, vomiting more than twice in the previous 24 hours, recent history of convulsion, unconscious state or inability to sit or stand) or signs of severe and complicated malaria in older children and adults, (iv) a pack cell volume (PCV) >15%, (v) absence of other febrile illnesses, (vi) written informed consent signed by the patient (for adults) or the parents or legal guardian (for minors), and, (vii) easy accessibility of their residence for home visits [26]. Before treatment, finger-prick capillary blood was collected on Whatmann 3MM filter paper for molecular analysis and in a capillary tube for PCV determination.

The co-blistered pack of AS + AQ (Arsucam^®^, Sanofi Aventis, Paris, France) containing 50 mg of artesunate and 153 mg of amodiaquine base per tablet was administered to patients under supervision. Patients received the standard daily dose of 10 mg base/kg body weight of amodiaquine and 4 mg/kg body weight of artesunate for three days. Based on the calculation using the body weight of patients, quarter, half, or three-quarter tablet was administered. For young children, the tablet was crushed and suspended in sugar-containing syrup. The patient was observed for 30 min. If vomiting occurred within 30 min following the initial drug administration, the treatment was repeated. The patient was excluded from the study if he or she vomited twice during the observation period.

The patients were followed on days 1, 2, 3, 7, 14, 21 and 28. Clinical examination and measurement of axillary temperature were performed during each visit, and any adverse events or unauthorized concomitant therapies were recorded. As recommended in the 2003 WHO protocol [26], blood films were examined on days 2, 3, 7, 14, 21, 28, and during any unscheduled visit if the patient became febrile. If parasites reappeared on or after day 7, finger-prick capillary blood was collected on Whatman 3MM filter paper for polymerase chain reaction (PCR) analysis.

The present study was reviewed and approved by the Congolese Ministry of Health. The WHO Secretariat Committee on Research Involving Human Subjects (SCRIHS) reviewed the study protocol and consent forms in French, English and local languages.

### Laboratory procedures

Thick blood films were stained with 10% Giemsa for 15 min. Asexual parasites were counted against 200 white blood cells (WBCs) and expressed as the number of asexual parasites/μL of blood, assuming a WBC count of 8,000/μL of blood. In case of hyperparasitaemia, the parasite count was determined when 500 asexual parasites were counted even if 200 WBCs were not reached. Parasite density was determined by two independent technicians. *Plasmodium falciparum* gametocytes were counted against 1,000 WBC. PCV was obtained by micro haematocrit centrifugation.

### DNA extraction and *Plasmodium falciparum* genotyping

Genomic DNA was extracted from blood samples collected on filter paper using QiaAmp DNA mini kit (Qiagen, Hilden, Germany) according to the manufacturer’s instruction. DNA was recovered in 100 μL of elution buffer. All extracted samples of parasite DNA were stored at −20°C until use.

For patients with treatment failure, the isolates collected at inclusion and recrudescent isolates were genotyped in parallel using nested PCR technique. The highly polymorphic loci, block 2 of merozoite surface protein-1 (*msp*-1) and the central region of merozoite surface protein-2 (*msp*-2), were used as markers for genotyping, as described previously [27]. The initial amplifications were followed by individual nested PCR using specific primers for K1, MAD20, and RO33 allelic families of *msp*-1 and specific primers for FC27 and 3D7 allelic families of *msp*-2. Allelic specific positive controls and DNA-free negative controls were included in each set of reactions. Five microlitres of PCR products were loaded on 2% agarose gel, stained with SYBR Green, separated by electrophoresis, and visualized under ultraviolet transillumination.

Individual alleles were identified by the fragment length and the corresponding allele-specific primers used, and the size of the PCR products was estimated using a 100 base pair (bp) DNA ladder marker (Invitrogen, Karlsruhe, Germany). The size polymorphism in each allelic family was analysed, assuming that one band represents one amplified PCR fragment derived from a single copy of *P. falciparum msp*-1 or *msp*-2 genes. Alleles in each family were considered to be the same if the fragment size were within 20-bp interval. The minimum number of genotypes per isolate was estimated to be the highest number of fragments identified for either *msp*-1 or *msp*-2.

It was assumed that after a patient was initially treated for malaria, a subsequent episode was caused by either *P. falciparum* isolates present before treatment (day 0) or by *P. falciparum* infections that occurred after treatment. First, paired pre-treatment and post-treatment samples were genotyped using *msp*-2 locus. If different band profile was found, it was concluded that the re-appearance of parasites on or after day 7 was due to a new infection. If similar profile of bands was observed, these samples were further analysed for a second locus, ie, *msp*-1. The outcome was defined as recrudescence if the paired samples (day 0 and recrudescent sample) displayed identical alleles. The outcome was defined as new infection if the recrudescent sample had only newly identified alleles. If the recrudescent sample showed new alleles and alleles identified on day 0, this sample was recorded as mixed infections or unclassified.

### Treatment outcome

The responses were classified before PCR correction and with PCR correction [28]. The PCR-uncorrected outcomes were classified into three categories: early treatment failure (ETF), late treatment failure (LTF), which consists of two subcategories, late clinical failure (LCF) and late parasitological failure (LPF), and adequate clinical and parasitological response (ACPR). PCR-corrected outcomes were classified into three categories: recrudescence, including ETF (on or before day 3) and recrudescence after PCR analysis, adequate clinical and parasitological response (ACPR), and new infections after PCR analysis.

### Adverse events

Signs and symptoms that developed after drug intake were observed and reported during clinical examination and questioning the patients or, in case of children, their parents or guardians, during each visit. Adverse events can also be an exacerbation of disease symptoms.

### Statistical analysis

All patients consulting one of the two health centres and satisfying the inclusion criteria were enrolled after written informed consent, regardless of their age. Two age groups were constituted: ≤5 years old and >5 years old. Moreover, the residence of the patients was classified as urban (patients consulting Tenrikyo health centre) or suburban (patients consulting Madibou health centre) area.

Clinical and parasitological data were entered into pre-programmed Excel spreadsheet provided by the Department of Global Malaria Programme, WHO (Geneva, Switzerland) with the possibility to analyse PCR-uncorrected and PCR-corrected responses. Proportions and 95% confidence interval (95% CI) were calculated using Epi-info 6.04 (Centers for Disease Control and Prevention, Atlanta, USA).

## Results

### Baseline characteristics of recruited patients

From February to August 2005, 1,087 febrile patients were screened for malaria in the urban Tenrikyo health centre, where 259 (23.8%) had a positive smear. During the same period, 161 patients were screened in the suburban Madibou health centre, where 76 (47%) patients were smear-positive. Of 1,087 patients, 264 (24.3%) received an anti-malarial treatment at home or in another health service. Monotherapies with classical anti-malarial drugs represented 89.8% of drug intake, of which >50% were chloroquine. Artemisinin derivatives accounted for 10% of drug intake (Table 1).

**Table 1:** Self-medication with anti-malarial drugs prior to consultation in Terinkyo and Madibou health centres in Makelekele district, Brazzaville

**Anti-malarial drugs**	**N (%)**
Patients	1,087
No self-medication	823 (75.7)
After self-medication	264 (24.3)
Classical monotherapies	237 (89.8)
Amodiaquine	38 (14.4)
Chloroquine	139 (52.7)
Quinine	42 (15.9)
Sulphadoxine-pyrimethamine	18 (6.8)
Artemisinin derivatives	27 (10.2)
Artesunate monotherapy	6 (2.3)
Artemether monotherapy	11 (4.2)
Dihydroartemisinin monotherapy	4 (1.5)
Artemisinin-based combination therapy (ACT)	6 (2.3)
Artemether-lumefantrine	1 (0.4)
Artesunate + amodiaquine	4 (1.5)
Dihydroartemisinin-sulphadoxine-pyrimethamine	1 (0.4)

A total of 197 patients were included from two health centres: 96 (48.2%) were children ≤5 years old and 101 (51.8%) were aged >5 years old (p >0.5) (Table 2). One hundred and twenty-five (62.8%) patients lived in the urban area of Makelekele district, while 72 (37.2%) resided in the suburban area of the district (p <0.001). The geometric mean parasite density in patients ≤5 years old (37,500 asexual parasites/μL) was higher than that of patients >5 years old (25,600 asexual parasites/μL) (p <0.001). The geometric mean parasite densities in patients residing in urban and suburban areas were similar, with 30,500 asexual parasites/μL and 30,520 asexual parasites/μL, respectively (p >0.5).

**Table 2:** Baseline characteristics of patients with uncomplicated falciparum malaria

	**Total**	**Age **		**Residence**	
		**≤ 5 years old**	**> 5 years old**	**Urban area**	**Suburban area**
Number	197	96	101	125	72
Mean age (years) ± SD	7.8 ± 9.3	2.6 ± 1.1	13 ± 11	7.2 ± 7.7	9.2 ± 11.7
range	0.7–54	0.7–4.8	5–54	0.7–52	0.7–54
Mean weight (kg) ± SD	22.0 ± 14.9	12.2 ± 3.1	31.3 ± 15.6	20.9 ± 13.0	24.6 ± 17.5
range	6–90	6–20	12–90	6–74	6–90
Sex ratio: Female/Male	94/103	42/54	52/49	64/61	30/42
Mean axillary temperature (°C) ± SD (range)	38.2 ± 0.78	38.3 ± 0.8	28.0 ± 0.7	38.2 ± 0.8	38.2 ± 0.8
	37.5–40.3	37.5–40.3	37.5–40.2	37.5–40.3	37.5–40.0
Mean geometric parasite density (asexual parasites/μL), range	30,800	37,500	25,600	30,500	30,520
	2,190–857,000	2,190–857,000	2,390–382,000	2,470–382,000	2,190–857,000
N of patients > 100,000 asexual parasites/μL	40	17	23	18	22
N of patients > 200,000 asexual parasites/μL	8	6	2	4	4
Haematocrit (± SD)	32.4 ± 5.6	30.8 ± 5.1	34.0 ± 5.4	32.4 ± 5.4	32.4 ± 5.9
range	15–50	15–43	15–50	15–50	15–46
Drug intake at home (Yes/No)	21/176	14/82	6/96	11/114	10/62

### Treatment outcome

During the 28-day follow-up period, 15 patients were excluded due to either lost-to-follow-up (n = 13, mostly in children aged <5 years old) or protocol violation (n = 2; unauthorized drug intake): two on day 1, three on day 2, three on day 3, three on day 7, one on day 14, and three on day 21). One 12-year-old patient with 30,600 asexual parasites/μL on day 0 presented asthenia on day 1. The attending physician considered that there was clinical aggravation and referred the patient for hospitalization. The outcome of this patient was considered as ETF. On day 1, eight of 197 (4%) patients were still febrile (axillary temperature ≥37.5°C). On day 2 and day 3, only two of 189 (1%) patients and one of 187 (0.5%) patients were still febrile, respectively. Parasite density decreased from the geometric mean (95% CI) 30,800 asexual parasites/μL (2,190 – 857,200 asexual parasites/μL) on day 0 to 235 asexual parasites/μL (49–3,700 asexual parasites/μL) on day 2. On day 3, only one patient had a positive smear at a low parasite density (315 asexual parasites/μL).

On day 0, six of 197 (3%) patients were gametocyte carriers. On day 2, day 3, day 7, and day 14, there were 16 of 190 (8.4%), 12 of 189 (6.3%), 11 of 187 (6%), and six of 185 (3%) gametocyte carriers, respectively. The highest mean gametocyte density, observed on day 2, day 3, and day 7, varied from 10 to 11 gametocytes/μL.

**Figure 1:**
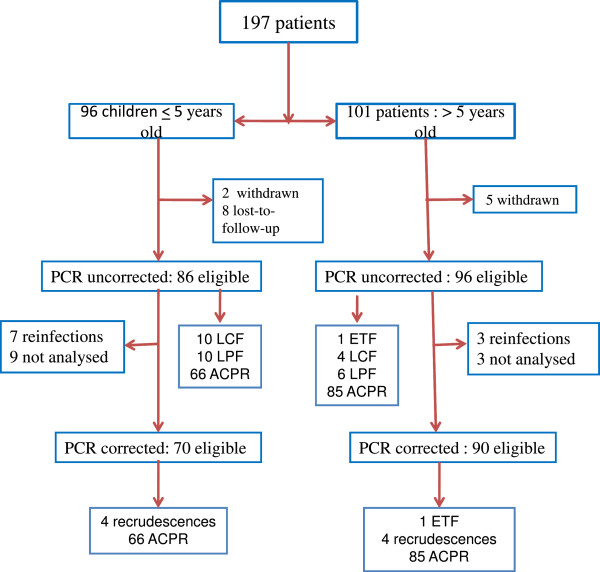
**Enrolment and follow-up profile.** Legend: ACPR, adequate clinical and parasitological response; LPF, late parasitological failure; LCF, late clinical failure; ETF, early treatment failure.

On day 28, the PCR-uncorrected cure rate (i e, the proportion of patients with ACPR) was 83% (151 of 182). In children ≤5 years old and children >5 years old and adults, ACPR were observed in 76.7% (66 of 86 patients) and 88.5% (85 of 96 patients), respectively (p = 0.07) (Figure 1). The proportions of ACPR in patients residing in urban and suburban areas were 84.2% (96 of 114 patients) and 80.9% (55 of 68), respectively (p = 0.5) (Table 3).

**Table 3:** Treatment responses on day 28

		**Total**	**Age**		**Residence**	
			**≤ 5 yrs old**	**> 5 yrs old**	**Urban area**	**Suburban area**
Number of patients: N		197	96	101	125	72
PCR uncorrected responses on day 28	Withdrawn + Lost-to-follow-up, N (%)	15 (7.6)	10 (10.4)	5 (5)	11 (8.8)	4 (8.1)
	Eligible; N (%)	182 (92.4)	86 (89.6)	96 (95)	114 (91.2)	68 (91.9)
	Failure; N (%)	31 (17)	20 (23.3)	11 (11.5)	18 (15.8)	13 (19.1)
	ETF; N (%)	1 (0.5)	0	1 (1)	1 (0.9)	0
	LCF; N (%)	14 (7.7)	10 (11.2)	4 (4.2)	9 (7.9)	5 (7.4)
	LPF; N (%)	16 (8.8)	10 (11.2)	6. (6.3)	8 (7)	8 (11.8)
	ACPR; N (%)	151 (83)	66 (76.7)	85 (88.5)	96 (84.2)	55 (80.9)
PCR corrected responses on day 28	Withdrawn + Lost-to-follow-up + reinfection + not analyzed; N (%)	37 (18.8)	26 (27.1)	11 (10.9)	24 (19.2)	13 (18.1)
	Eligible; N (%)	160 (81.2)	70 (72.9)	90 (89.1)	101 (80.8)	59 (81.9)
	Failure; N (%)	9 (5.6)	4 (5.7)	5 (5.6)	5 (5)	4 (6.8)
	ETF; N (%)	1 (0.6)	0	1 (1.1)	1 (1)	0
	Recrudescence; N (%)	8 (5)	4 (5.7)	4 (4.5)	4 (4)	4 (6.8)
	ACPR; N (%)	151 (94.4)	66 (94.3)	85 (94.4)	96 (95)	55 (93.2)
	Recrudescence; N	8	4	4	4	4
	Reinfection; N	10	7	3	3	7

*N*, Number; *ACPR*, adequate clinical and parasitological response; *LPF*, late parasitological failure; *LCF*, late clinical failure; *ETF*, early treatment failure.

After PCR adjustment, the overall treatment efficacy was 94.4% (151 of 160 patients responding with ACPR). In children ≤5 years old and those aged >5 years, the proportions of ACPR were 94.3% (66 of 70 patients) and 94.4% (85 of 90 patients), respectively (p = 0.5) (Figure 1). There was no significant difference (p < 0.05) in the proportions of ACPR in patients residing in the urban (96 of 101 patients [95.0%]) and suburban (55 of 59 patients [93.2%]) areas, respectively. The risk of treatment failure was not different between children aged ≤5 years and those aged >5 years, including adults (p = 0.6), and between patients living in urban and suburban areas (p = 0.4).

### Clinical adverse events

The frequently reported adverse events by adult patients and children aged >5 years, as well as by the parents or legal guardians of young children aged ≤5 years included dizziness (15%), vomiting (14.6%), diarrhoea (9%), pruritus (9%), headache (7%), anorexia (1.5%), and abdominal pain (1.5%). Most of these signs and symptoms disappeared on day 3. In a few cases however, headache and pruritus persisted until day 7. On day 1, two patients were excluded due to repeated vomiting.

## Discussion

The present study was conducted to provide supporting evidence for the clinical efficacy of AS + AQ, which was adopted in the new anti-malarial drug policy in central Africa, including Congo, to replace ineffective, classical anti-malarial treatment based on chloroquine and sulphadoxine-pyrimethamine [9]. Despite their low efficacy, these monotherapies had been frequently prescribed in health services and widely available and taken for self-medication at home for febrile episodes during the period when the present study was conducted.

This is the first clinical study on ACT conducted in Brazzaville, where 37% of Congolese population resides. A single earlier clinical study on AS + AQ and artemether-lumefantrine was conducted in a rural area [22]. However, the present study has several weak points: it is non-randomized and non-comparative, and patients of all ages were included. The latter weakness was corrected by analysing and comparing the data according to age strata (≤5 years old *vs* >5 years old, including adults).

The results of the present study show that AS + AQ reduces fever very rapidly. More than 95% of the patients were afebrile on day 1, ie, after the first dose of treatment. The geometric mean initial parasitaemia decreased rapidly after only two doses, and 99% of the patients had negative smears on day 2. There were only a few gametocyte carriers during the follow-up period. Other clinical studies in Africa have already highlighted these properties of AS + AQ on fever, parasitaemia and gametocytes [29-31].

The overall efficacy of AS + AQ after PCR correction, represented by the ACPR rate, was 94.4%. The results of the present study are in agreement with those of the earlier study in a rural zone in Congo, in which a cure rate of 98.5% was reported (n = 66) [22]. If the efficacy observed in patients within the same age range (i e, ≤5 years old) in both studies is considered, efficiency was similar (p = 0.36).

Studies conducted in Central Africa before or during the same period as the present study used the non-fixed formulation of AS + AQ (Arsucam^®^). The reported efficacy was excellent in one randomized study conducted in Angola with 100% of ACPR [32]. In the Democratic Republic of Congo, two studies conducted between 2003 and 2004 in Equator, South Kivu, and Katanga provinces reported paradoxical results with 85%, 93%, and 100% of ACPR, respectively [33,34]. In two studies conducted in Gabon, AS + AQ has shown an efficacy of 90% [29] and 86% [35]. The efficacy reported in the present study is comparable to that recently reported in other African countries, like Ghana [36,37].

Many of the adverse events reported by the patients and parents or legal guardians of young children were probably caused by amodiaquine, as described in many previous studies on amodiaquine monotherapy. Although these adverse effects were qualified as minor, they may be one of the causes of poor compliance if AS + AQ treatment is left unsupervised.

A new fixed formulation of AS-AQ with three dosages (25 mg/67.5 mg, 50 mg/135 mg, 100 mg/270 mg), which is available today, reduces the total number of tablets for the three-day treatment regimen and improves patient compliance. Since 2008, all these dosages have been available free of charge in the Congolese public health sector as first-line anti-malarial.

## Conclusions

The present study demonstrated AS + AQ efficacy in Brazzaville using non-coformulated ACT. The present results serve as a database for further clinical evaluation to determine therapeutic efficacy and tolerance of fixed AS-AQ formulation in the capital city of Congo.

## Abbreviations

95% CI: 95% confidence intervals; ACPR: Adequate clinical and parasitological response; ACT: Artemisinin-based combination therapy; ETF: Early treatment failure; LCF: Late clinical failure; LTF: Late treatment failure; PCR: Polymerase chain reaction; SCRIHS: WHO Secretariat Committee on Research Involving Human Subjects; WHO: World Health Organization.

## Competing interests

The authors declare that they have no competing interests.

## Authors’ contributions

MN, PIM, PNC, and DL performed the clinical study. MN, LKB, FN and PB performed and validated the data analysis and wrote the manuscript. All authors read and approved the final manuscript.
